# Picking the Right Parts at the Beta Barrel Factory

**DOI:** 10.1371/journal.pbio.0040399

**Published:** 2006-11-07

**Authors:** Mary Hoff

Tube-shaped protein constructs known as beta barrels are used by Gram-negative bacteria as passageways for molecules that need to travel from one side of the outer cell membrane to the other. Getting beta barrels embedded into the membrane is a daunting task because of the nonpolar nature of the membrane’s insides; the way cells manage that is with the assistance of a tubular factory already embedded in the membrane that takes in and assembles the outer membrane proteins (OMPs) that comprise the beta barrel. A protein called Omp85 is a central component of this factory in a surprising number of bacterial species as well as in mitochondria and chloroplasts (eukaryotic organelles that arose from bacteria).

To learn about the structure and function of this evolutionarily conserved part of the beta barrel factory, Viviane Robert, Jan Tommassen, and colleagues reconstituted *Escherichia coli* Omp85 in vitro, then looked at how it interacted with the OMPs that assemble to form beta barrels.

When placed in an artificial membrane, the reconstituted Omp85 formed a voltage-activated channel—a property that could be used to study its interaction with substrate OMPs. Indeed, when the researchers exposed their construct to denatured OMPs, they found an interaction reflected by increased Omp85 channel activity, presumably to begin the beta barrel assembly process. But they also found that it didn’t interact with other proteins they tested. What, they wondered, is the secret password OMPs use to access the Omp85 factory?

Previous studies had shown that a “signature sequence” of amino acid residues on the C terminus of OMPs—consisting of a phenylalanine or tryptophan at the very end, and hydrophobic residues in the 3, 5, 7, and 9 positions from the C terminus—is important to cell survival in bacteria that use Omp85 to make beta barrels. That led the researchers to hypothesize that the signature sequence is key to gaining entry into Omp85. To test their hypothesis, they looked at a mutant OMP that lacked the proper C-terminal signature sequence. The mutant OMP not only was not admitted by the Omp85, but at high concentrations, it even blocked the Omp85 channel completely. In further support for their hypothesis, the researchers manufactured an imposter—a synthetic peptide with the appropriate C-terminal signature sequence—and presented it to the Omp85. As they predicted, it stimulated the channel to open, providing additional evidence that Omp85 indeed recognizes OMPs by their C-terminal signature sequence.

**Figure:**
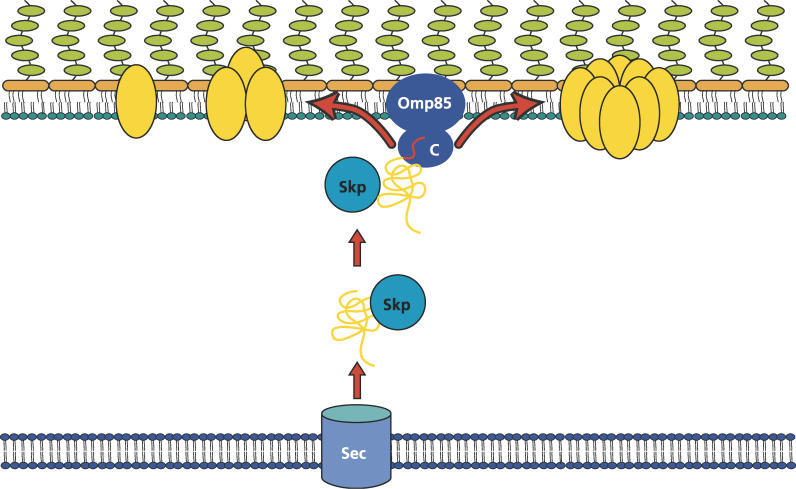
Planar lipid bilayer experiments revealed that the evolutionarily conserved assembly factor Omp85 recognizes the signature motif present at the C termini of bacterial OMPs.

Since Omp85 is found in a variety of bacteria, the researchers decided to test whether the C-terminal signature sequence key from one species would unlock the Omp85 gates in another. They found that neither PorA (an OMP from *Neisseria meningitidis*) nor its C-terminal peptide pushed the right buttons for *E. coli* Omp85, even though their C-terminal signature sequence is similar to that of *E. coli* OMPs. This fits with previous observations that the presence of *N. meningitidis* OMPs is fatal to *E. coli*, but it also raises the question as to what the discriminating characteristic might be. To find out, the researchers compared C-terminal sequences of *N. meningitidis* and *E. coli* OMPs. They discovered that *N. meningitidis* OMPs tend to have arginine or lysine residues at position 2 from the C-terminus, while *E. coli* OMPs do not. Further testing of OMPs with various amino acid residues in the penultimate position provided further support for their speculation that that particular residue is responsible for the species specificity they observed.

The researchers concluded that the use of an Omp85 factory to get OMPs into the outer membrane is conserved across species, but some differences in recognition of appropriate OMPs have evolved since the organisms evolutionarily diverged. As a result, Omp85 can selectively exclude not only non-OMPs, but also OMPs from other sources as it goes about its business of building beta barrels.

